# Sentiment and Science*Presidential address at 37th annual meeting of the State Medical Association of Texas, Houston, Texas, April 26, 1905.

**Published:** 1905-05

**Authors:** F. E. Daniel

**Affiliations:** Austin, Texas


THE
### TEXAS MEDICAL JOURNAL.

ESTABLISHED JULY, 1885.

PUBLISHED MONTHLY.—SUBSCRIPTION $1.00 A YEAR.

VOL. XX. AUSTIN, MAY, 1905. No. 11.

### Sentiment and Science.*

BY F. E. DANIEL, M. D., AUSTIN, TEXAS.

Intellectual man is the full-blown blossom of organic evolution, and its crowning glory. Evolved through countless ages and myriads of forms — from the protozoa through the invertebrates and the vertebrates—he reached his physical supereminence, science tells us, only a comparatively short while ago,—some fifty million years, perhaps, when, abandoning his arboreal habits, he walked erect and became—man.

The dawning of a moral sense in man is still more recent, and only in the more advanced races may it be said, has it developed into what we call a moral nature; the knowledge of right and wrong; good and evil; for we see it in the process of evolution, from a faint suggestion of an awakened sense, through barbarous and semi-barbarous races, to civilized man, with whom it is the governing law, and upon which are founded social law, religion, and ethics.

By heredity, even civilized man retain to this day of his physical, intellectual, and moral supremacy, traces more or less pronounced of his brute origin and instincts. Not only physical, as the nails, the canine teeth, the appendix vermiformis, but the desire, propensity and will to fulfill the laws of his animal nature, and gratify his selfish wants without regard to the rights of others.

Primitive man just emerging from the brute state, was unrestrained by any social or other laws. He knew nothing of the "rights of others," nor of the rights of property. These are con-

*Presidential address at 37th annual meeting of the State Medical Association of Texas, Houston, Texas, April 26, 1905.

cepts that have grown out of the social order for the protection of the weak and the peace of society. "Others" had no rights which he felt bound to respect. Impelled by the laws of his nature, he took what he wanted, if strong enough to take it,—food, raiment, weapons, and the females of his kind. It is contrary to the laws of man to do these things. To violate the laws is a crime for which society punishes him. When we speak of a moral law, we have in mind the higher order of man,—the finished product of evolution, —for there are still races so low in the scale, who inhabit New Caledonia, Africa, India, Asia, and Australia, and other parts of the earth, who have no moral sense, who live in trees, as the ape men of India, lying on beds of branches, as do the orang-outang and the chimpanzee; who have no articulate language, as the aborigines of Borneo (Buchner); who abandon their young and the aged to starvation; who know not the use of fire, but eat their food raw; who have (Lindsay) no sense of decency, modesty, chastity, virtue, propriety, shame; no love, maternal, paternal, conjugal, filial or fraternal; no idea of paternity or other relationship; no kindness to, or consideration for, each other; no compassion, pity or sympathy for suffering; no mercy, no regret, remorse or repentance; no gratitude; no sense of guilt or criminality; in short, no moral sense whatever, and in some cases, as in that of the savages in N'Yanza (Baker), "not even a superstition on which to found a religious belief," but who practice the reverse of all that we consider moral and virtuous and deem it right. It is only the more highly organized who are evolved out of the state through which these races are passing, that have a moral sense and feel responsible to a moral law. That there should remain in him even now traces of his animal nature, pure and simple, and which we regard as the evil or "bad" in man, is natural and true. It is the hereditary transmission of these things that lingers in man and constitutes the bad principle; education and evolution have not yet totally eradicated it, even in the highest type of man, else we would have no crime and criminals, and no need for jails, prisons, and punishments.

Hence, with the growth of that moral sense which distinguishes civilized man, there began, and is now, and ever has been, a conflict between the "good" and the "evil" of his nature.

From the earliest ages these two conflicting and contending principles have been recognized, and are typified by the early and crude concepts of "Deus" and "Theus," Jove, Jehovah, Allah, God,—and "Satan," "Asmodeus," "Apollyon," "Lucifer." These concepts stood for and stand for the "good" and the "bad" in man. Milton makes very real the conflict between them, and "Paradise Lost," next to the Bible, has done more to emphasize and perpetuate this figure of speech than all other agencies combined. The belief in "good spirits" and "guardian angels," and "evil spirits," and "angels of the Prince of Darkness," is universal. There are

"Spirits to bless and demons to damn."

We see it in the philosophy and religion of all races, Christian and heathen. In some it takes the form of tutelary deities, as with the Japanese and Chinese, who worship the spirits of their ancestors, —a belief transmitted from the myths of prehistoric civilizations. In the Greek and Persian mythologies the good-spirit idea is typified by the Lares and Penates, guardians of households, individuals and communities. Of these, frequent mention is made by Virgil, Cicero, and Ovid. The complement of that belief and the antagonist, is typified by the omnipresent "Devil," the genius of evil, contending ever for the possession of men's souls. In Stephenson's classical allegory, "Dr. Jekyl and Mr. Hyde," the two principles, good and evil, are presented in a way that strongly contrasts them, and it is shown how, by the cultivation of the evil, the good may be dwarfed until the nature of man becomes all bad. When the moral sense is totally absent or in entire abeyance, we have a criminal, judged by our standards.

The principle of "good" is both intellectual and moral: the "bad" is that instinct just alluded to, inherited from our pithecanthropoid progenitors. In this conflict is embraced also the question of right and wrong; but I will not enter upon a discussion of that phase of the subject, because there is no fixed standard of right and wrong for the regulation of conduct that is universally recognized, or applicable to all peoples, because of the different degree of development as stated; and differences of opinion as to what is right and what is wrong have occasioned most of the wars and trouble of the world.

The concepts of these principles are the results of heredity, environment, education and custom, and, of course, they vary greatly. What is held to be right by one people or in one age, may be, and is, in another age and with another people regarded as wrong and vicious. I instance polygamy, upon which I need not enlarge, and consanguineous marriages. They are prohibited by law in most civilized countries, experience having shown that the latter tends to race suicide, and the former to a low state of morals, destructive of the foundation stone of society,—the home and the family.

In approaching my subject, therefore, I will confine myself to a consideration of that ever present, never ending conflict between the two elements of "good,"—the intellect and the emotions—the realistic and the ideal; if you please, between the rational, real, positive, the empirical, on the one hand, and the emotional, the visionary, the sentimental, the transcendental, on the other; the conflict between Knowledge and Belief, between Science and Sentiment.

Embraced under the term "sentiment," are all the virtues of both head and heart. It is beautiful and ennobling. It makes one's life sublime. It prompts to all good and noble thoughts, deeds and acts. It is sentiment that controls the conduct of all good men and gives them their real character. It alone brings comfort in life, solace in adversity, and hope at the hour of death. It embraces the virtues of pity, mercy, love, affection, kindness, charity, friendship, hope, faith, reverence, religion,—and that sympathy a touch of which makes all the world akin. It is belief, transcendentalism; faith, as opposed to empirical science (knowledge), and is represented by man's emotional nature. Sentiment gives us all the poetry and romance of life. It scatters flowers along the pathway and makes life's road less rugged. It is to man what the perfume is to the flower. It furnishes the theme of the poet, the painter, the sculptor, and is the basis of our polite literature—song and story. A man destitute of these principles is a monster. In our schools and by every hearthstone the cardinal virtues of pity, mercy, and charity are, or should be, inculcated. They make a man's real character and stamp the seal of a community's morals. Cruelty to the humbler animals, the brutes, and the helpless and unfortunate of our kind are evils that are not, and should not be, tolerated.

It always seemed to me strangely inconsistent that parents and teachers who are kind-hearted, tender and merciful, and who love their children and desire of all things that the best in their natures shall be developed, make the mistake of whipping them—whipping them for being "bad." Not vicious or debased, but "*bad.*" They fight, they climb trees and tear their clothes, they throw rocks, they play hookey, they tramp in mud, they go swimming contrary to orders,—maybe go fishing on Sunday. For this they are whipped. It is brutalizing. It crushes the pride and manhood out of a boy. Every blow upon a child's back leaves a scar on his soul. He will falsify next time to avoid the cruel lash. It makes liars and thieves of your boys. A mother can control her boy and make sure of his love and obedience by tact and affection, and I want to say to parents here tonight, the mother-influence goes with a boy through life and moulds his character and shapes his conduct more than any other influence. It is natural for a boy to be "bad." It is his animal nature; pent-up energy that must find vent in overt acts. A "bad" boy is a natural boy, a healthy boy. A "good" boy is pathologic. He's an invalid.

Healthy sentiment is beautiful. But there is apt to be degeneracy here as elsewhere, into a fanaticism that will carry one to extremes. Reason, neglected, will be dwarfed, and kept in abeyance. Such fanaticism for centuries prevented the progress of science in any direction, and held the world in the bonds of tradition and superstition. Everything inconsistent with the creeds and traditions of ecclesiasticism was "heresy," for which there was but one treatment. The brightest minds were extinguished in the fires lighted by fanaticism. I need only to hint at Bruno, the long line of middle-age Popes, and the "gentle Torquemada," the Grand Inquisitor. Visions of the terrible *auto da fé*, almost daily demonstrated, and the anathema of "heresy" had a powerful restraining influence on those who dared to think, and dampened their zeal and ardor in the cause of natural laws against fable and tradition. Sentiment, degenerated into fanaticism, led Peter the Hermit to devote to destruction forty thousand men, women, and children in the first crusade, and Godfrey, Louis IX of France, and Richard of the Lion Heart, and Frederick of Germany to later deluge the world in blood, and the bones of the fallen crusaders were sacred and were carried home for interment. This sentiment attaching to the remains, led Shakespeare to cause to be engraved on his tomb a curse upon him who should disturb his bones.

It is necessary that "evil" shall be done; "evil," from the standpoint of the emotional, that permanent good may result. When the surgeon finds it necessary to inflict pain,—to cut out a tumor or to amputate a limb to save life,—is it to be said that he is "cruel," and that he feels not the human sentiment of pity and sympathy? Pity for the sufferings of the humble, the helpless, the unfortunate, is one of the noblest sentiments that can actuate the human heart. It leads to practical charity and prompt to those acts of kindness which so distinguish the civilized and enlightened. Margrave, the man without a soul, destitute of pity or remorse, the central figure in Bulwer's "Strange Story," supposes a purely intellectual man, one in whom there is no conflicting emotion or sentiment, and he is a monster. The fanaticism, on the other hand, that leads the hysterical and super-sentimental to look upon vivisection, for instance, as *all* evil, and who can not see that, like the surgical operation just instanced, it is necessary in order that good may follow, represents the other extreme. In them there is no conflict between head and heart; it is all heart. In their minds there is no counteraction of reason. It is in abeyance or undeveloped. This fanaticism leads to the writing of such books as "Black Beauty," and "Trixy," admirable lessons, but extreme and fanatical, and to the organization of anti-vivisection leagues and crusades against vaccination and cruelty to animals, carried even to the prohibition of laboratory researches in comparative physiology and physiologic chemistry. The sufferings of the poor dumb brutes is pictured so vividly as to horrify the reader and awaken sentiments of disgust for the heartless scientist who would dissect a living sentient creature. Is it not to be supposed that these investigators have hearts in their bosoms, and deplore that it is necessary? men whose every heart-throb is for humanity, and who do these things, not that they do not love all of God's creatures, and sympathize with their sufferings, but because they love their fellow-man more?

In earlier times even dissecting the dead human body was prohibited, because, at the resurrection day the body should be whole, the ecclesiastics said.

The State of Texas in this, the Twentieth century, founded a great medical college, and educates her young doctors free. A professor of anatomy and an assistant are paid large salaries to teach anatomy. It costs $50,000 a year to maintain the college. The State requires its graduates to stand examination in anatomy (the basis of medical education), for the degree, and again for license to practice; yet our laws make it a felony to procure the material for the study of anatomy. The faculty of the medical department of our great university, through the State Medical Association of Texas, asked the Twenty-ninth Legislature to legalize dissecting corpses of the paupers who die in our charity institutions, when unclaimed by friend or relative after publication. The bill was not treated with the respect due to any request coming from such a source, especially one in the interest of medical science and the public health, but was ridiculed outrageously; literally laughed out of court. It is almost incredible, but it is true. Educated gentlemen, dignified Senators, treated, the request with a contempt, and a distinguished Senator, who last session was the protagonist for legitimate medicine, wanted to amend the bill so that it should apply only to the corpses of doctors. "Let them dissect each other," he said. Such sentiment prevailed in the Dark Ages, but is most surprising in this enlightened age, and is a disgrace to the State. It was not until the days of Wharton Jones (1840-2), whose pupil was the great Huxley, that physiology was even differentiated from anatomy, and histology was unknown. Nothing was known of the vital processes of the human body, of excretion, secretion or digestion; metabolism was not dreamed of; nothing was known of the functions of the glandular system or the nervous system; nothing of neurons, not even of cells, until within the last half century; and nothing was known of the mind, absolutely nothing. It was regarded as synonymous with the soul, and was a hypothetical and intangible something, outside of and independent of the brain, and which exerted an influence on the life and controlled the actions of man, determining his fate as to hereafter.

All, or nearlv all, that is known of the mechanism of life was learned by experimentation on the lower animals. Only as late as 1851 Claude Bernard discovered the great vaso-motor function of the sympathetic nervous system, and he did it by dissecting a turtle.

"Waller and Brown-Sequard had previously shown that stimulation of the peripheral part of the divided sympathetic constricted the blood vessels and reduced temperature, but Bernard's experiment marks the beginning of our vaso-motor knowledge." (Sir Michael Foster.) Thus was a knowledge of the mechanism revealed, whereby the distribution of blood supply is regulated, and an explanation afforded why a man may not bleed to death in his own veins. "The Webber Brothers, by vivisection (1845), ascertained that an electrical impulse sent down the vagus nerves will stop the heart from beating; while impulses reaching the heart along the cardiac sympathetic nerves from the thoracic spinal cord stir up the heart to more vigorous and frequent beats. Hence, our first conception of inhibition (ibid). Both a check and a spur to the great central organ, the heart, were placed in man's hands.

The effects of chloral hydrate, paraldehyde, sulphonal and other similar drugs were all discovered by experiments on animals. * * * In the case of people who, from wakefulness through nervous irritability are actually on the high road to insanity, these drugs have been of extreme value. Salicylic acid,—almost a specific in rheumatism,—strychnine, which enters now into about one of ten of the prescriptions written by doctors, is of inestimable value in nervous exhaustion and atonic dyspepsia. Nitrate of amyl in the horrible convulsions of uremia, and epilepsy, and in the agony of neuralgia of the heart; ergot, to the use of which thousands of mothers owe their lives; digitalis, for feeble heart action, the doctors describe as literally "snatching people out of the grave,"—all discovered by laboratory experimentations on animals; and carbolic acid, discovered by Lamaire, and introduced in surgery by Listér, has removed the horrors and reduced the mortality of operative surgery of earlier days, by preventing blood poisoning, tetanus, fever, secondary hemorrhage, and gangrene, all of which used to prove so fatal (The "Professor," Holmes).

Indeed, it would be impossible to enumerate the thousands of benefits that have come to medical science in life-saving agencies through such investigations. To mention only anesthesia by chloroform, cocaine, ether; vaccination, antitoxines, the discovery of the cause of consumption by the immortal Koch, will convey an adequate idea. We will sum up the gross results in the last sixty years, accruing to man through medical and sanitary science: In 1840 the average length of life was thirty-three and one-third years; it is now 48.6. Thus, life has been lengthened 50 per cent—fifteen years added to the life of every person.

Says the veteran sanitarian, Dr. A. N. Bell (*Stamina, Medico-Legal Journal,* December, 1904):

"The chief increase has been during the latter half of that period, and, for the most part, by the reduced mortality from zymotic diseases, but, above all, from pulmonary tuberculosis, from which the reduction of mortality has been nearly 50 per cent. This reduction has been effected, almost wholly by sanitary efforts; by dealing with and destroying unsanitary surroundings, soil-drainage, purifying water supplies, reporting and restricting communicable diseases, sanitary supervision of schools, the destructon of sputum—the now everywhere recognized fountain-head from which the army of bacilli is perpetually reinforced—abolishment of cellar-dwellings, diminished overcrowding, cleanliness, disinfection, isolation and aeration; improved tenements, opened-up and wider streets, public parks and recreation grounds and establishment of sanitaria."

There are still very many problems yet to be studied and solved in the biological laboratory. There are animals whose economy does not react to certain poisons as does man's. Why? Why will one poison or a particular grouping of chemical molecules "act" on one nerve and not on another? Why will one arrangement of the molecules of the same chemical spur the heart (as atropine, $C_{17}$, $H_{23}$, $N.O._3$), while a nearly precisely similar grouping will show it (as homatropine—$C_{16}$, $H_{21}$, $N.O._3$)? Cocaine ($C_{17}$, $H_{21}$, $N.O._4$), will completely destroy sensation in a part if injected under the skin, without interfering with consciousness, while morphine ($C_{17}$, $H_{19}$, $N.O._3$, $H_2O$) will cause unconsciousness.

Says Prof. Chittenden, of Yale: "There is something marvelous in the unerring certainty with which a given group of cells (glandular) performs its work, never deviating a hair's breadth from the beaten course, and turning out year after year a definite line of products for the specific purpose in view. Why is it that the epitherial cells of the salivary glands always manufacture mucinogen and ptyalin; the gastric gland cells pepsinogen, renninogen and hydrochloric acid; the cells of the pancreas trypsinogen and steapsin; the hepatic cells bilirubin, biliverdin and the specific bile acids; the cells of the thyroid iodothyrin, and the cells of the adrenals epinephrin? Essentially the same blood and lymph bathe all these cells with a like nutritive pabulum, and yet each group of cells performs its own line of work, never going astray, in health, and never even temporarily producing a product which rightfully belongs to the other class of cells."

These secretions all are concerned in and are essential to digestion, nutrition and metabolism, and they are all elaborated from the blood, the product of the food that sustains and perpetuates life. "The discovery of the secretion of glycogen by the liver," says Sir Michael Foster, "came as a bolt from the blue."

It is impossible to estimate the bearings of these problems and discoveries on medical science and their value to mankind.

Yet all of our studies and inductions in comparative physiology and physiologic chemistry are, and must be, on the lower animals, and some of these problems can never be worked out, unless the day shall come when an enlightened public sentiment and the law shall devote to science the condemned criminal, say the negro sadist, the black peril that casts its shadow over our fair land, more baleful than the lethean shade of the deadly Upas. Not to dissection, as people understood it, but to experimental study on the internal organs to solve the problems of immunity, fermentation and glandular action.

Sentiment revolts at the suggestion. But, regarding him,—the condemned perpetrator of the nameless crime as a waste product,—the scum in the ebulition of the social cauldron, why not make use of him for the advancement of medical science and the interests of that world he has outraged and forfeited? After having served the purpose of investigation and experimentation to determine some of the problems that can only be solved by studying the economy of a human being, he could be put to a speedy and painless death by simple means. During the investigation he would be anæsthetized as far as possible. Cocaine injected into the investing membrane of the spinal cord will produce anesthesia of the abdominal region and lower limbs, without destroying consciousness or interfering with secretion, excretion, glandular action, or any of the vital processes. Chloroform or ether will produce total anesthesia — a dreamless, tranquil sleep. Why not?

Would such utilitarian process be more shocking or cruel than the tortures inflicted in our reformatories and prisons for some infractions of prison discipline, too horrible to relate here, but which are common and well known? Or than the brutal gallows or electric chair? Would it be worse than war, wherein, not criminals, but the flower of the youth and chivalry of the land are torn by shot and shell and sabre stroke, or trampled, broken and bleeding, out of all semblance of humanity by the hoofs of charging squadrons of cavalry? The Russians dig pits into which the charging hosts of the enemy fall by the hundreds and are impaled alive on spike of steel, or fall on oil-soaked straw, which is fired by an electric spark on contact, and burned alive. The world tolerates such brutality and calls it "glory."

True, two wrongs do not make a right. War is horrible beyond compare, and most revolting. It is "wrong," but it seems to be inevitable and necessary; and in the upward evolution of nations, is probably beneficial. Vivisection is a necessary evil, that permanent good may result. Wanton and purposeless cruelty, or cruelty for sport, is to be condemned by all persons, of every shade of opinion or belief. Bull fighting, cock fighting, trap-shooting of live birds are species of cruelty that an enlightened community should not tolerate. It is the survival of the savage in us, the hereditary brute instinct—blood-lust. An ex-President of the United States, in a magazine article describes with an almost audible smack of the lips, the delight of duck killing, and says that if he fails to bring the bird to bag, it is a satisfaction to know that he went off "lead-loaded," that he had given him a "buck dose." I wonder if he ever gave a second thought to the wounded bird, and realized that hidden in the grass or weeds—an easy prey to its enemies—it suffered an agonized, tortured and lingering death? I confess that reading that article did not increase my respect for the ponderous and pompous duck-shooting statesman.

Let us not talk of cruelty to animals, and sicken at the thought of utilizing, in the interest of the advancement of science, for the promotion of human happiness and welfare, a waste product of human evolution; let us not strain at so small a gnat so long as we have the brutal sports, and that largest size camel—war—forced down our throats.

The discovery of the function of the white corpuscles of the blood, the great "eating cells" that constitute the army of defense in the human system, and which overpower and destroy invading disease germs, would never have been made but for laboratory experimentations. It led to the discovery of toxines, thrown out by the invaders, and of antitoxines, the result of the bacterial warfare, whereby not only the pathogenic germs are overcome, but the blood is left in a state that renders another invasion powerless, a state of immunity to that disease. This discovery gave us the cue to the production of antitoxines in the laboratory with which to reinforce the army of occupation in its fight with disease germs; hence, serum therapy—the greatest advance in medical science in the century. By repeatedly inoculating the horse with culture of the diphtheria bacillus, the horse's blood becomes the scene of this warfare, and soon is rendered immune. The serum of this blood is the curative and preventive and immunizing agent that has robbed of its horrors diphtheria — the Moloch to which so many children were sacrificed. Prior to this discovery, a few short years ago, fifty-four children out of every one hundred died when attacked with diphtheria, and the infection was spread to every child of the family or of the neighborhood. Now, less than 1 per cent die if promptly subjected to the influence of this God-given agent, and every child in the family and in the community can be protected from the infection by inoculation.

Think of it, ye sympathetic brothers and sisters who would have Congress prohibit laboratory investigation out of consideration of the sufferings of Black Beauty or of Trixie or Fido, losing sight of the merciful sleep brought to them by chloroform or ether, and of the life-saving agencies thus put in the hands of the medical profession. Bring it home to your children, mothers, fathers. You have four, from the cute little babe who, with its two little milk teeth, playfully bites when drinking at the fountain of delight, and looks up with a roguish smile to see if mama can take a joke; Johnny, in his first knickerbockers, a curly-headed, ruddy cheek, black-eyed rowdy, who breaks things, makes a noise and tramps mud on your clean floors,—papa's chum and partner; Susie, aged 10, mama's darling, who sits by her side and learns to sew; and Carrie, the firstborn, a maiden of sweet sixteen. She is budding into womanhood, and

"Standing with reluctant feet
Where the brook and river meet"—

the dawn of the lovelight in her eyes, like the first breath of perfume in the opening bud, foretells the glories of a splendid womanhood, "as fresh and as sweet as a Mermet rose kissed by the morning dew." Ah, how you love them! They are the tie that binds your hearts together and gives to life its greatest charm. Carrie has sore throat and is sent home from school. The doctor looks long and carefully at her throat. He removes something on a probe and examines it under a microscope.

"Yes, my dear madam, it is diphtheria."

"Oh! oh! my pets! my darlings! Save them, doctor, save them! My blue-eyed girls, my black-eyed boy, my baby"!

Mother, what were the lives of a thousand Black Beautys and Trixies and Fidos; what were all the horses and dogs in the world to the light in the eyes of those precious ones—the laugh and noise of that boy?

Be consoled, dear, distressed mother. Science, the Delilah, has shorn this Sampson of his strength; Science, the tiny David, has overthrown this great monster, Goliath; Science, like Perseus to the rescue of Andromeda chained to the rock to be devoured by the fabulous sea monster, can rescue your precious ones from the jaws of Death.

Say, ye fanatical sentimentalists who would have Congress turn back the clock of time to the dark ages, when the insane,—diseased human beings,—were chained in dark dungeons, beaten and starved, would you spare a few rabbits and dogs and turtles and frogs, and reinvest diphtheria, bubonic plague, hydrophobia, with all their horrors?

Bring it home to yourself again: Your son Jack, aged 18, a fine, manly fellow,—"just like his papa,"—is your hope and stay. In your old age you will lean on Jack,—you and dear old mother. You idolize him. He is in your office. He relieves you already of the cares of your business to a great extent. Horrors! Jack is bitten by a mad dog! You see, as swift as the wings of light, your boy in the agonies of hydrophobia. In your frantic anticipations and excited fancy, you see him in convulsions, foaming at the mouth, with staring eyes bursting from his terror-stricken face, and you and he and mama realize and sicken at the thought, that he must surely die a horrible, lingering death!

Be not alarmed, dear father and mother. Those days have passed away. Such cases died before God inspired the immortal Pasteur. In ancient times he would have been deified, and translated to the skies, become the brightest constellation in the galaxy of light and glory. Surely it was inspiration. Pasteur made a culture of the bacillus of rabies and inoculated a rabbit; a poor, scared, timid little bunny, with eyes soft and tender and a silky coat. See how her little heart beats; hear her feeble cry. Pity, pity, yes, pity a thousand times that it must be so. The spinal cord of that little rabbit is the God-sent weapon with which our modern doctors can save Jack. Would you exchange one hair of Jack's head for all the rabbits on earth?

Habits and customs, the outgrowth of sentiment, often inimical to health and happiness, have been transmitted through generations and are so deeply fixed in the human heart and mind that reason will never be able to uproot them. The object of interment of our dead is to dispose of the remains,—that its elements may be resolved back to earth again, yet our methods of interment defeat the end, and the dead are a menace to the living. Sanitary science,—in the interest of the living,—sharing not at all in that unhealthy sentiment that attaches almost universally to the human dead, proposes a sensible "safe and sane" substitute for it,—incineration,—whereby those horror-haunted cities of the dead may be abolished, and graveyards be made a thing of the past. Sentiment still clings to the mouldering past, and opposes sanitary reform. Sentiment prompts to the gloomy and depressing wearing of mourning for the dead,—too often an outward symbol of a grief not felt,—a lie and a hypocritical pretense, but too often, also, a sincere expression of that grief that time alone can assuage. It is a survival of the sack-cloth and ashes, and is as senseless. Sanitary science condemns it. It is useless, unbecoming, expensive, unreasonable, unhygienic, and to be condemned from every standpoint. A crusade should be preached from every pulpit against the custom of wearing black, and every physician should urge its abolition.

Science is organized knowledge in action. It is empirical, in the sense that knowledge is the accumulated experience of the world. Man can know nothing but that which comes to his consciousness through his senses, and he can not understand anything which can not be compared to or contrasted with something within human experience, and so, symbolized in his mind. Hence, the effort to symbolize God results in the anthropomorphic conception of the Deity. Belief is transcendental; it goes beyond experience. It needs not, asks not, proof or evidence. Science demands both, and rejects that which is contrary to reason and human experience. Hence, the ever-widening gulf between science (reason) and tradition (superstition, belief, faith); not between science and true, real, *religion,* for there is not and can not be any conflict between them. Every advance of science, the unlocking of every secret of nature, every interpretation and revelation of her laws that comes to man through scientific research, proves, confirms and stamps with the seal of eternal truth the existence of the Supreme Intelligence,—the Great First Cause,—the soul and source of all life, energy and intelligence.

Hence, science has its limitations. There are things that man can never know. Sydney Lanier, seeing a mocking bird catch and devour a grasshopper, exclaimed:

"Solve me this problem, ye scientists:
How is the death of that dull insect
Become the life and song of yon trim
Shakespeare on the tree"?

"What is the shaping power behind the egg? (cell)," asks Lowell.

Back of all the phenomena of the universe there are only two elementary and fundamental things: matter and motion (energy). All that we see consists in changes in the one or the other of these two forms. This is the postulate of experimental science. (Daster.) All the phenomena of life can be explained upon the principle of chemical action whereby the force or energy which resides in matter—all matter—in a latent or potential state is liberated or converted into active (kinetic) energy. This, the new science of energetics teaches, and more; a science born of the immortal conception, the grandest inspiration that ever came to the human mind, that of an obscure young physician of Wurtemburg, Robert Meyer. He first conceived of and announced the conservation of energy and the correlation of forces. The great Helmholtz took it up and actively supported it, fixing it firmly in the scientific mind, by his well-known essay on the subject. Some fifty years previously (1778) Lavoisier had announced the doctrine of the indestructibility of matter. "Nothing is created, nothing is lost. All is change," and "chemistry is the history of the mutations of matter." These doctrines form the basis of the science of energetics, which brings all phenomena, including the vital, under the empire and dominion of the physical laws of the universe, and accounts for all phenomena without invoking any external or supernatural agency.

Scientific thought has ever had a predilection for the "mechanical" view of life, and I may say that it is now accepted by all properly informed persons (outside of the theological schools, of course) who have given the subject proper and careful consideration. It has superseded, displaced and relegated to the domain of exploded theories the long held, stoutly defended and greatly cherished dogma of "animism" or "vitalism"—doctrines (purely superstition) based on "belief"—unsupported by any evidence.

The question of life, of mind, of consciousness, is not transcendental. It is purely a physiological problem, partly already solved in the biological laboratory. When solved it will be found to fall within the domain of physics and chemistry, and come under the laws that govern molecular mechanics. Life of all animals is now known to be evolved within the body by chemical action.

I need not say that all energy is derived from the sun, nor more than allude to the reciprocal functions of animal life and plant life. Plants store the sun's energy and we find it after centuries, still stored in the coal we dig from the bowels of the earth, where it has remained in a latent or potential state. Plants split up the carbon dioxide of the air, freeing the oxygen, man's vital necessity, and that of all breathing animals, and store the carbon in wood, starch and sugar. It is the action of oxygen on the carbon, whether in coal or wood or in our own aliment (essentially carbon and nitrogen compounds) which liberates this energy, converting it from the latent or potential state into active (kinetic) energy. This, in the animal economy, is subdivided, or specialized, and expressed in the various well-known forms of "radient" energy, "thermal" energy, "kinetic," or moving energy, "chemical" energy and "electric" energy (including magnetic). These forms are all correlated, and are intro-convertable, transformable, the one into the other, except heat energy. Heat is not transformable directly into any other form of energy. Take the locomotive for an illustration. The combustion of the coal put into the furnace (a chemical process, oxidation) liberates the potential energy stored in the coal by the sun. It is expressed in those modes of motion (molecular) known as heat and light. The heat changes the molecular arrangement of the water in the boiler, converting it into an expansive vapor, which, being confined, seeks to escape, and thus drives the piston, which moves the engine and train (mechanical energy, kinetic energy). The steam also propels a dynamo, whereby a part of this liberated energy is "specialized" and transformed into electric energy, which is expressed in the headlight that blazes on the locomotive's front, and in the incandescent light, whereby the coaches are lighted.

All the mechanical contrivances of man, the pump and valves, the lever, the arch, the pulley, were copied by man from God's model and masterpiece,—the human machine. In the invention of our complicated electric system of telegraphy, telephony, and heating and lighting, man has unconsciously duplicated in a crude way, the nervous system of man and the higher animals; the wireless telegraphy, I believe, is identical — mechanically — with what we are bound to recognize as thought-transference, — telepathy. The brain is a living dynamo for the transformation of energy into electricity.

Man's energy (his life force) and that of all animals, is derived from the food put into his stomach and from the reserves of nutrition already stored in his tissues. All food that has nutritive value, is essentially carbon and nitrogen in endless combination. The liberation and conversion of this latent (potential) energy is effected by chemical means (oxidation, combustion), and it is expressed in all the phenomena associated with the life of the animal—heat, muscular motion (kinetic energy, mechanical or "work" energy); secretion, digestion, assimilation, metabolism, destructive and constructive, * * * and *consciousness*, the fundamental state upon which rests the mind, with its multifarious phases or functions of thought, volition, ideation, all the higher intellectual faculties. Consciousness is a part of the higher activities of the mind (soul?), and as such is dependent on the normal integrity of the psychic structure, the brain, and on a continuous supply of energy; of oxygen, of fuel, of food. Cuvier calls the human system a "vital vortex," through which there is an endless circulation of matter and energy from the outside world, by ingestion, secretion and excretion. How consciousness arises no one knows any more than he knows how life began, what gave to "matter" (protoplasm,—the primitive cell) sensation, motion, and I am sometimes disposed to think, intelligence. But it is doubtless a product of the food taken into the stomach. It is a part of the life energy liberated by the chemical reaction between the carbon compounds of the aliment (and the tissue reserves) and the oxygen taken in through the lungs, and is specialized or told off for special work by the wonderful electro-chemico dynamic apparatus we call the nervous system, properly the net work of special cells (neurons) that ramify in and connect every part of the body. If anyone doubt that consciousness and all mental states are the product of the food and air, let him withhold all food, and see how long it will last. The mind in all its phases, is the function or product of the brain. It is a force, a specialized force, and is electrical. We see, hear, feel, think and act by electricity. The touch, the thrilling kiss is an electrical discharge. This force, the mind, can be directed by the will. It can be directed towards a person, say, across a hall or church or theater, and that person be made to feel it, precisely like the artificial electricity projected through space by the Marconi system (waves—vibrations), can awaken a response in a receiver attuned to the proper wave lengths. It will awaken a sleeping person to gaze at his face, and we all know how an eloquent speaker can sway men's thoughts and move them to overt acts,—even to commit violence.

The mind, then (and when I say mind, I mean all that is understood by the term), is electrical energy, generated by chemical action, and is purely mechanical. It is a mode of motion generated by a living organ,—the brain,—the original dynamo, and as surely ceases when the combustion which produced it ceases, when the brain dies, as does the flame of a lamp when the oil gives out. Science can as easily understand and as readily believe that the shut-off light of an incandescent lamp, exists and persists, for all time, as the special and individual and separate light,—an entity of itself,—as that a life force, the mind, generated in precisely the same way (combustion,—oxidation of carbon), and transformed and specialized into the electrical form of energy can continue after the death of the organ, and consequent cessation of the processes by which it was produced. It is impossible to think of mind disassociated with organization.*

Indeed, the tendency of modern scientific thought is to reduce all matter and energy to electricity,—to the point where the material and the immaterial blend so completely as to be undistinguishable, —the *identity* of matter and energy; and yet, no one knows just

*I have quoted at some length from my paper "Natural or Supernatural" in *Medico Legal Journal*, December, 1903.

what electricity is, or can define it better than by saying it is a form of energy. On the last analysis, according to the new electric theory of matter, matter is electricity, and the basis of all matter and energy is the monad,—electrified units,—compared to which the now discarded atom, a quite complex body, is as this building to a grain of sand. Matter (mass), it was held, may be divided and subdivided till it reaches the vanishing point; hence science hypothecated the atom (no one ever saw one) and for years it was held to be the starting point of all matter. The postulate that "thoughts are things," once considered so absurd, seems not so inconceivable after all. "The universe is God's thought." Mr. Balfour, premier of England, and president of the Royal Society, in his presidential address before the society, recently said:

"But the electric theory which we have been considering carries us into a new region altogether. It does not confine itself to accounting for the secondary qualities by the primary, or the behavior of matter in bulk by the behavior in atoms; it analyzes matter, whether molar or molecular, into something which is not matter at all. The atom is now no more than the relatively vast theater of operations in which minute monads perform their orderly evolutions; while the monads themselves are not regarded as units of matter, but as units of electricity; so that matter is not merely explained, but is explained away."

The conflict between sentiment and science, between knowledge and belief, between fact and fancy, was waged through all the centuries from Aristotle and Plato to Huxley and Haeckel, Newman, Gladstone, Wilberforce,—reason fighting a long time against fire, sword, and blood,—narrowed down in the nineteenth century to the question of evolution as against tradition. Darwin was not the originator of what is understood as evolution, but Lamarck. He lived in a time when such doctrines were regarded as "heresy," for which there was but one remedy. Hence, it was not developed until the days of the younger Darwin, and would have fallen flat but for the championship of the "strong man of science," Thomas Huxley. He became the protagonist of evolution, and overthrew all debaters pitted against him,—Gladstone, Newman, Wilberforce,—routing the latter, horse, foot and dragoon in a memorable debate in 1875. Tradition went down in defeat, and evolution is forever fixed in the scientific mind as the correct interpretation of God's eternal law and order.

Everything conceivable, organic and inorganic, in the great universe of God is evolved from a starting point. From the tiniest seed to the magnificent flower with its dazzling tints and its breath of intoxicating perfume; from the embryonic cell, the germ plasm of the human being, to the finished product, moral and intellectual man; from nebulous masses of "cosmic matter," to blazing suns, there is birth, growth, equilibrium, decline, decay, death, in everything, individuals, races, nations, worlds, systems and the universe. Look into the heavens and observe, in the great nebulæ of Orion and Andromeda, the process of sun-building always going on; the birth of new worlds from the germs of extinct worlds that have finished the cycle of evolution. They were born, grew, developed their maximum of life, declined, decayed, died; and their elements are entering into the construction of new worlds, perhaps of a higher order. It is the same with individual man, and with races and nations. From the parent cell of ancestors, the individual man is evolved, attains his adult existence, develops the best that is in him (perhaps), decays, and passes away, but lives again in his descendants. Under God's law, unhampered by man's mistaken sentiment of humanity (humanity to the individual—inhumanity to the race), the fittest survive. All our humanitarian work, pity for the defectives, fosters the survival of the unfit, by the perpetual propagation of the defectives, which, left to the operation of natural law, would be eliminated. That sentiment that leads us to put no restrictions on marriage is the most potent factor in the swift degeneration of the race; it is race suicide. The population of Texas in forty-five years, to date, increased 504 per cent. The insane in Texas in the same time increased 6800 per cent, a ratio of nearly 14 to 1. Criminals in America (United States census, 1900) increased at a ratio of 3½ to 1 of population. In Texas the insane, the criminal, the pauper, the consumptive, the scorbutic, the syphilitic, are permitted to marry, with the sanction of the law and the blessing of the church. Aye, even the blind, and the deaf mutes. That is sentiment. Science would insist upon stirpiculture—the rational propagation of the human species—else, the fittest can not survive. Lydston, in his recent epoch-making book, "Diseases of Society," says that a feeble dude with a monocle in his eye gazed through the bars of a pasture upon the splendid proportions of a magnificent bull, and, addressing the bull, said: "By Jove, old fellow, you are a splendid specimen of your kind, don't you know?" The bull replied: "I'm sorry, my son, that I can't say the same for you; you are a miserable specimen of yours, don't you know? But I'll give you a pointer: If the same pains and care had been taken in the selection of your ancestors that were taken with mine, you would not have been the miserable little failure that you are, don't you know?" or words to that effect.

It is sentiment that endows man with a soul. Dr. Wm. Osler, now Regius professor of medicine at Oxford, and the greatest living authority on medicine in the world, perhaps, says (Ingersoll lecture, 1904): "Modern psychological science dispenses altogether with the soul. * * * The association of life in all its phases, with organization,—the association of a gradation of intelligence with increasing complexity of organization, the failure of the development of intelligence with an arrest in cerebral growth in the child, the slow decay of mind with changes in the brain, the absolute dependence of the higher mental attributes upon definite structures, the instantaneous loss of consciousness when the blood supply is cut off from the higher centers,—these facts give pause to the scientific student when he tries to think of intelligence apart from organization." * * * "The old platonic and orthodox view has no place in science, which ignores completely this something that will be, after us. The new psychologists have ceased to think nobly of the soul, and even speak of it as a complete superfluity."

Science, though a benefactor to the race, is yet a brutal iconoclast. With club and rapier, it hewed its way to the light of truth through a wilderness of superstition, tradition, fiction, fable, and unsupported beliefs in things scientifically impossible; and the flowers of fancy that embellish the literature of the world are ruthlessly scattered. Science says that angels are impossible; and evolution says that there was never an Adam. What would the world be without the beautiful story of Adam and Eve and the Creation? Science now relegates it to the domain of fable along with so many other cherished and delightful creations of the mind of faith and fancy, and would uproot so much that is held to be sacred and true by the great mass of the people. With ruthless hand, science would even tear away the foundation upon which is erected the great superstructure of the most beautiful and ennobling belief, by denying all miracles in general and the possibility of human parthenogenesis in particular. The physical laws of the universe are immutable and can not be reversed or suspended. God is the soul and source of those laws, and He never contradicts himself. (This is the "heresy" for which Urban VIII burned Bruno.) Sentiment adheres to the miracle of human parthenogenesis—the foundation of the dominant religion of the world. It could only have occurred by miracle, and science maintains that there is not another recorded instance in the history of the world. There are many instances in fable, but none in fact. Parthenogenesis occurs amongst insects (heterogamy), notably the bees. The workers (the females) are legitimate children—all princesses, daughters of the queen; but the males, the "drones," one of which must become the father of the next generation, are a sort of by-product, and have no paternity. But nothing of the kind has occurred amongst men since the days of Jupiter. Horace tells us that in those days Jupiter, in the form of a shower of gold, descended through the roof of the tower where she was confined, and into the lap of Danæ, and became the father of Pereus, for instance, a warrior of great renown and valorous deeds. At death he was translated to the skies, and we see him nightly in all the glory of the great constellation that bears his name. That branch of science we call psychology—the science of the mind (soul)—would take even a rationalistic view of the Apocalypse and of St. John's beautiful vision of the Holy City with its walls of jasper, its streets of gold and its gates of pearl, and with cold-blooded calculation would bring the visions of both Saul and John within the category of well-known psychoses.

But sentiment still adheres to tradition and superstition. It is the popular and prevailing belief, taught from thousands of pulpits and the altars of prayer in the households of millions of intelligent, earnest, good people; and it is so firmly and deeply fixed and rooted in the human heart that science can not shake it. The arrows and missiles from the quiver of science glance harmless from the battlements as from walls of adamant; for back of all the "energy," and "science," and "physical laws"; back of all the phenomena of human life and of the universe there is something, something intangible, inscrutable, infinite and incomprehensible, that can not be revealed by microscope or scalpel, nor weighed nor estimated. We must fall back upon Sentiment and Faith at last, and call that something the Great First Cause, and bow in humility and reverence before the universal manifestation of His might, majesty and power! For, however reason would persuade us to the contrary, there is something away down in the depths of the heart and the inner consciousness of every rational creature that tells us, "It must be so, else why this pleasing hope, this fond desire, this longing after immortality?"

Let it be false or true. Let it be knowledge or belief, fact, fancy, faith or fable, it is pleasant to think that there are angels that guard us, and at death waft on spirit wings the souls of the pure in heart to that land of promise of which our mothers told us. It is consoling and sustaining to believe and to hope that our dear ones departed are there, and that we shall meet them. Who knows?

"O, if perchance there should be a sphere,
Where all is made right which so puzzles us here,
Where the glare and the glitter and tinsel of time
Fade and die in the light of that region sublime;
Where the soul disenchanted of flesh and of sense,
Unscreened by its trappings and shows and pretense,
Must be clothed for the life and the service above
With purity, truth, faith, meekness and love,"—

man could have no higher, purer, nobler aspiration than the hope inspired by this belief; and though reason says "No, it can not be," the eye of Faith catches the glint and gleam of celestial robes, the ear of Hope hears the rustle of angel wings, and I am ready to exclaim with Cicero:

"I would rather be wrong with Plato than right with those who refuse to the yearning soul the balm and consolation of this blessed hope."

